# Navigating life after laryngectomy: a qualitative study on adjustment and distress in the first 6 months following surgery

**DOI:** 10.1007/s00520-026-10525-0

**Published:** 2026-03-12

**Authors:** Penny Chapman, Karyn Galvin, Katrina Blyth, Jemma Skeat

**Affiliations:** 1 Speech Pathology Department, St. Vincent’s Hospital Melbourne, 14 Nicholson Street, Fitzroy, Victoria 3065 Australia; 2 University of Melbourne, Melbourne, Victoria Australia; 3 University of Sydney, Sydney, Australia; 4 Deakin University, Melbourne, Victoria Australia

**Keywords:** Adjustment, Cancer, Distress, Laryngectomy, Support, Oncology

## Abstract

**Background:**

Total laryngectomy, the surgical removal of the larynx, is a life-saving procedure for individuals with advanced laryngeal or hypopharyngeal cancer. While the physical consequences of this surgery are well documented, the psychological, emotional, and social adjustment to life post-surgery is less well understood. The aim of this study was to explore the lived experience of adjustment within the first 6 months following a laryngectomy.

**Methods:**

A longitudinal multimethods study was undertaken at St. Vincent’s Hospital, Melbourne, Australia, involving patients scheduled for total laryngectomy between August 2018 and April 2020. Data were collected in one-to-one, in-depth, semi-structured interviews at 2 weeks post-hospital discharge and at 3 and 6 months post-surgery. Psychological distress was assessed preoperatively and prior to semi-structured interviews, using the Distress Thermometer. Interview data were analysed using inductive thematic analysis.

**Results:**

Four participants completed all three interviews, and a further two participants completed one interview each. Three phases of adjustment were identified: (1) *Not normal life*, (2) *Never going to be the same again*, and (3) *Just get on with it*. Emotional distress was moderate 2 weeks following hospital discharge but reduced significantly by 6 months post-surgery.

**Conclusion:**

This study sheds new light on the lived experience of adjustment following total laryngectomy, highlighting a clear pattern of psychological, emotional, and social transition in the acute healing period. The findings underscore the importance of timely, multidisciplinary support that aligns with patients’ evolving needs. Understanding the phases of adjustment provides clinicians with a framework to proactively guide and personalise care during this vulnerable period.

## Introduction

 A total laryngectomy operation involves the surgical removal of the larynx for management of advanced laryngeal or hypopharyngeal cancer. A total laryngectomy operation is life-changing, resulting in significant physical and functional changes affecting breathing, verbal communication, and swallowing [1, 2]. A total laryngectomy also results in significant psychological and social changes [3, 4].

For individuals who require a total laryngectomy, the diagnosis of cancer coincides with the news that a significant life-saving operation is necessary. This means that they, along with their family, must quickly come to terms with the impending removal of their larynx, which will significantly impact communication, swallowing, and breathing and result in a permanent tracheostoma. The window between receiving a laryngeal cancer diagnosis and laryngectomy surgery is often less than 2 weeks apart, due to airway compromise, particularly for individuals with advanced laryngeal or hypopharyngeal cancer [5].

Preoperative education for a total laryngectomy focuses on preparing patients and their family for the long-term adjustments post-surgery [6] and typically involves screening for psychological distress. It is crucial to provide education about the physical and functional consequences of the surgery, both before and after the operation. Additionally, support to help patients adapt to the psychological effects is essential, as difficulties in adjusting to cancer treatment can lead to anxiety, depression, and distress [7].

Psychological distress related to total laryngectomy surgery has been found in 20 to 25% of patients in the preoperative period [8]. A peak of anxiety preceding a laryngectomy and a peak of depression within the first 3 to 6 months has also been identified [4]. Health care professionals working with patients who are candidates for a total laryngectomy should ensure that social and psychological changes are addressed in the preoperative and post-treatment education [8].

Managing post-laryngectomy changes is a unique and individualised experience. Some studies have highlighted the major adjustments required by individuals in managing the life-changing effects on breathing, communication, swallowing, and altered body image [9]. Many psychological symptoms have been reported following laryngectomy, including anxiety, depression, suicidal thoughts, fear of recurrence, loss of self-esteem, and uselessness [4] Total laryngectomy can affect one’s communication in a variety of settings which may be isolating and may lead to disruptions in mealtimes with family and friends and impact one’s socialisation with others [10]. Having a permanent tracheostoma alters or reduces a person’s self-identity [11] as well as their ability to perform activities of daily living and participate in leisure activities [12].

Qualitative research focuses on individuals’ experiences and can capture critical moments and processes involved in change, particularly adjustment [13]. Current qualitative studies exploring adjustment experiences of individuals post-laryngectomy have focused on longer term adjustment beyond 12 months post-surgery. It has been reported that individuals greater than 12 months post-laryngectomy experience an altered self-identity [14]. A loss of self-expression was reported as being more than just changes to verbal communication, but also included altered appearance due to a tracheostoma, reduced capacity to eat or manage in social occasions when eating out, and changes to social and work roles [14]. Dooks et al. [9] explored the perspectives of people 6 to 12 months post-laryngectomy. They found that having an altered airway was one of the day-to-day challenges that participants reflected on. They also noted the immense impact an altered airway had on their lives, from management of secretions to coping with changes to communication and in emergency situations. The loss of voice, combined with altered body image, was identified as an immense adjustment to cope with in everyday life [9].

At present, there are no qualitative studies that explore adjustment experiences of individuals post-laryngectomy in the acute phase. Such research is required in order to understand the factors that contribute to or impede early adjustment and to illuminate individual experiences and coping strategies in the first 6 months post-surgery. Therefore, the aims of this study were to explore (1) distress levels and (2) adjustment experiences of individuals within the first 6 months following laryngectomy surgery.

## Methodology

This study was approved by the St Vincent’s Hospital Human Research Ethics Committee (Reference Number: HREC/18/SVHM/187). The research was conducted in accordance with the Declaration of Helsinki.

### Study design

A longitudinal multimethods design was chosen for this study. The quantitative approach, using The Distress Thermometer, allowed researchers to determine levels of distress and problems identified over the 6 month period. The qualitative approach, using semi-structured interviews, allowed the researchers to gain a deeper understanding of the phenomenon of early adjustment experiences. The longitudinal design, involving three interviews over 6 months, enabled exploration of adjustment trajectories over time and captured critical moments and processes involved in change [13].

### Participants

Potential participants were all patients 18 years or older who were planned for a total laryngectomy at a tertiary hospital in metropolitan Melbourne between August 2018 and April 2020. Patients with a diagnosis of dementia or a significant cognitive impairment (as determined by their treating medical/allied health team) were excluded from the study. The invitation to participate was provided by an on-site independent speech pathologist, with informed consent obtained by the same speech pathologist prior to participants undergoing surgery. All six patients planned for total laryngectomy surgery were notified of the study by an independent clinician. All six consented to participate in the study.

### Measures

#### Distress

**Figure. 1:**
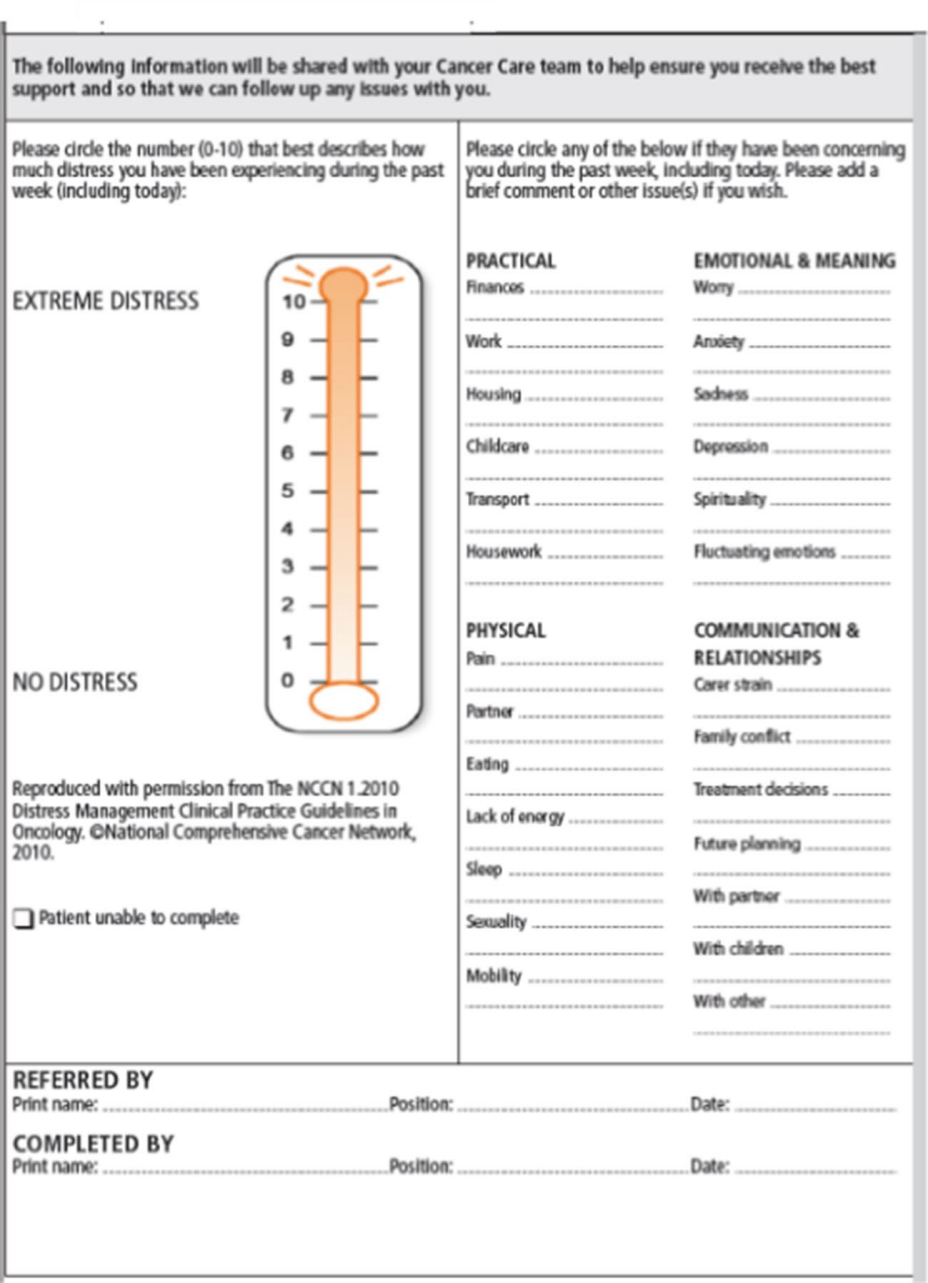
Distress thermometer and problem checklist

Participants completed the Distress Thermometer and Problem Checklist (see Fig. 1) prior to their surgery as well as at 2 weeks following hospital discharge, and at 3 and 6 months post-surgery. The Distress Thermometer was developed by Roth et al. [15] to measure an individual’s distress level prior to and following cancer treatment. This screening tool takes approximately 2 minutes to complete and comprises a visual analogue scale from 0 (no distress) to 10 (extreme distress). The problem checklist comprises (1) practical, (2) physical, (3) emotional and meaning, and (4) communication and relationship domains to determine the respondent’s supportive care needs [16].

### Adjustment experiences

One-to-one semi-structured interviews were conducted with each participant at 2 weeks post-hospital discharge as well as 3- and 6 months post-surgery. Each interview was conducted by the primary author (PC) using the same topic schedule (see appendix). The interviews were conducted either in a private and quiet room at the treating hospital or in a private telehealth session and lasted up to 40 minutes. Participants used a mixture of tracheoesophageal speech (TEP speech) and writing. All interviews were audio and video recorded.

Interpretative rigor, triangulation (including methods triangulation, triangulation of sources, researcher triangulation), member checking, and rigorous reflexivity were employed to ensure quality in research processed.

#### Data analysis

Inductive thematic analysis was undertaken by two researchers (PC, JS) following the six phases described by Braun and Clarke [17].

1. *Familiarising yourself with the data*
2. *Generating initial codes*
3. *Searching for themes*
4. *Reviewing themes*
5. *Defining and naming the themes*
6. *Writing the report*

Specific quotes representing themes and concepts were utilised to support an understanding of the adjustment experiences of participants. In order to understand the longitudinal aspect of the data, analysis took place both within each data collection period and across data collection periods, examining the process of adjustment over time. The themes developed from both the interviews and the Distress Thermometer tool provided the researchers with an interpretative description or a reframing of the phenomenon experienced by people post-laryngectomy. 

## Results

### Demographic information

A summary of each of the six participants is presented in Table 1.

**Table 1:** Demographic, hospital stay, surgery, co-morbidity, and family support information for the six participants

Participant	Age (yrs)	Gender	Residence Area	Languages spoken	Hospital LOS# (days)	Surgical details	Medical background	Family support
P1*	79	M	Metro	Italian & English	20	Salvage laryngectomy partial pharyngectomy, ALT free flap TEP & voice prosthesis	SCC epiglottis & chemoradiotherapy, GORD, ex-smoker	Y
P2	77	F	Inter-state	English	29	Salvage laryngectomy, partial pharyngectomy, ALT free flaps × 2, TEP & voice prosthesis	SCC supraglottic & radiotherapy, SCC lung & surgery, ex-smoker, COPD	Y
P3	81	M	Metro	English	38	Salvage laryngectomy, partial pharyngectomy, ALT free flap, TEP & voice prosthesis	SCC larynx & chemoradiotherapy, GORD, COPD	Y
P4	72	M	Rural	English	19	Salvage laryngectomy, TEP & voice prosthesis	SCC larynx & radiotherapy, GORD, ex-smoker	Y
P5**	51	M	Metro	English	15	Salvage laryngectomy, TEP & voice prosthesis	SCC larynx & radiotherapy, ex-smoker, T2DM	Y
P6	66	F	Metro	English & Arabic	15	Total laryngectomy, TEP & voice prosthesis	Drinks alcohol, ex-smoker, depression	Y

# Length of stay; * P1 death at 3 months post-surgery; **P5 withdrew from study at 3 months post-surgery

*TEP* tracheoesophageal puncture, *ALT* anterolateral thigh, *SCC* squamous cell carcinoma, *GORD* gastro-oesophageal reflux, *COPD* chronic obstructive pulmonary disease

### Thematic analysis

**Figure. 2:**
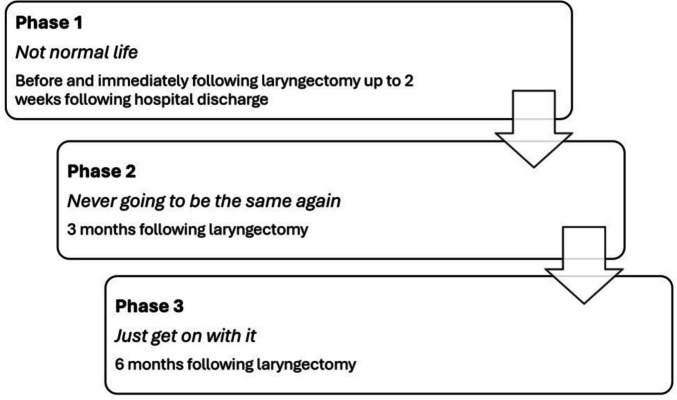
Phases of Adjustment during the first 6 months

Thematic analysis revealed a rich description of the participants’ adjustment experiences following laryngectomy. None of the six participants received psychology services, as this resource was not available during the study period. Within and across the data collection periods, there were three phases of adjustment that participants went through during the first 6 months: Phase one: Not normal life; Phase two: Never going to be the same again; and Phase three: Just get on with it (see Figure. 2).

As shown in Table 2, there were moderate levels of distress in phase one and little or no distress was seen over time.

**Table 2:** Distress thermometer scores for each participant at 2 weeks post discharge and at 3 and 6 months post-surgery where 0 is no distress and 10 is extreme distress

Participant	Pre-op score	2 week D/C	3 month	6 month
1	0	0	No score	No score
2	4	4	3	0
3	0	0	0	0
4	5	5	0	0
5	4	4	No score	No score
6	6	0	0	0

*Participant 1 died before the 3 month screening**Participant 5 withdrew from the study before the 3 month screening

D/C = hospital discharge

#### Phase One: Not Normal Life (Pre-operatively, during admission and following hospital discharge)

An early point of adjustment for participants was related to the upcoming surgery and accepting that they were to undergo a laryngectomy. Participants needed to adjust to the idea of what they would lose by having the surgery; this adjustment occurred pre-operatively, often quite close to the diagnosis of their cancer. Participants needed to make a decision regarding proceeding with the surgery; given the information they had been provided with by their medical team, they viewed the surgery as life-saving. Participants had some idea that they would ‘lose normal life’ through the surgery, but reflected on the decision to proceed with surgery as one made in order to live for their family:

"I didn’t want to do it at first but if the cancer was going to kill me, I then agreed to the operation…my thoughts were around my family." P6

At this stage, moderate levels of distress were experienced by four participants regarding the surgery and its life-changing nature. The preoperative Problem Checklist section of the Distress Thermometer identified the concerns participants held at the time of diagnosis. Physical concerns, such as pain, sleep, eating, and lack of energy, were commonly identified by participants. Psychological concerns, such as worry, depression, anxiety, and sadness, were highlighted and significant. Participant four shared that they contemplated suicide:

"All I wanted to do was commit suicide. I got in my ute one day and I was going to put it up against a tree but I didn’t do it." P4

The time in hospital post-surgery played a role early on in this adjustment phase. Being in hospital was not normal life. Participants reported a lack of control, a lack of normal life routine, a negative impact on mental health, physical disfigurement, delayed healing, and functional restrictions. Their description of their hospital stay revealed what factors can reduce the capacity to cope and negatively impact adjustment. Some negative experiences are highlighted below.

"Too long. My hospital stay kept getting extended. I was frustrated. I didn’t enjoy being locked up. I felt like I couldn’t go anywhere, this was it." P3

"I felt like I was in hospital too long and I missed everyone at home and I missed my dogs." P6

During this time of ‘not normal life’, support from the hospital staff was important to the participants. The support they received and the kindness and positivity they experienced assisted the participants in adjusting. The care and support provided by nurses, doctors, and allied health staff were pivotal in aiding adjustments in altered breathing, voicing, and swallowing:

"The nurses were kind and caring. The nurses and staff were nice to everyone. I felt comfortable and safe. It was a nice atmosphere. The staff were like family." P6

Returning home was described by participants as a pivotal point in their adjustment, as home represented a sanctuary, freedom, and a return to a more normal routine. At home there was no pressure to do personal activities of daily living compared with hospital. Participants reported that they felt free at home to do what they wanted and they also felt a sense of relief to be out of hospital:

"It was great returning home as I had no pressure. No pressure to get moving or get dressed or have a shower. I could go at my own pace at home." P2

Despite feeling more free at home, participants’ early-stage post-discharge experiences also highlighted their new dependence on others, for example, in relation to their tracheostoma care and their voice prosthesis. It also highlighted the importance of ongoing support that they received from others. Participants discussed how much support they received from family or friends and how crucial this was to their adjustment to this new not normal life:

"My neighbour helps me, she visits each day. The nurses come to my home twice a week. I always have good help." P2

#### Phase Two: Never Going To Be The Same Again (3 months post surgery)

Participants moved from recognising their loss of normal life and towards an understanding that life is never going to be the same again. While the initial phase explored above had been a time of rapid change, in Phase 2, participants were making longer term adjustments to living with their laryngectomy and the ongoing implications it held for their life. There was little to no distress reported by participants during this phase. They were starting to question how they would manage the changes to their life and starting to accept these changes as part of their normal routine. They were realising further limitations that were perhaps not so obvious during Phase 1; these included not being busy, having reduced fitness, being dependent on others, having an altered swallowing, and having compromised communication with others.

"Life is shit. I feel like I’m the only person in the world that has had this surgery. When I go to town there’s no one else that looks like me. People look at me and question what is wrong with me. I feel like a freak." P4

"You don’t have normal interactions with everybody because you don’t talk normally. You’ve got to clean the bloody mucous out all the time." P3

Participants discussed the physical impact of the surgery on their altered self. This included struggling to talk with others using tracheoesophageal speech, coping with an altered swallow, mucous production from the tracheostoma, breathlessness, and reduced fitness levels. One participant described himself as feeling handicapped:

"I feel handicapped. This bloody hole has been worrying me. I get out of air…Breathless…I’ve always had a very active life but after this operation I’m not as active as I was… my lungs get affected… A good day is when I don’t have much phlegm. I reckon it’s getting less and less…maybe life is a little better…I’m trying to keep busy." P4

Participants recognised things they missed about life before the operation. One particular area was their voice. Participants described that their altered and compromised voicing with tracheoesophageal speech was a daily reminder that life was never going to be the same again:

"I miss calling out to the cattle, I can’t yell. They don’t respond to my voice." P4

"I miss my voice. I can’t argue with my husband. He doesn’t hear my voice or understand it and he gets angry and then I get angry." P6

It was also during this phase that participants identified what factors supported their adjustment; these factors included the importance of being in their home environment, support from others, keeping socially connected, and aiming for independence.

"You’re just in charge of your own life when you are home. My kids visit me and keep in touch. Their support is helpful as it provides a connection with people and they help me with things." P3

"I use the computer to email family and I use Skype to call the family. I use my iPhone too to facetime my daughter. The technology helps me with communication. I don’t feel so isolated. I feel isolated from my sisters in NSW and QLD." P2

Another important concept voiced by participants was the value of education and training. Education and training were provided in hospital by nursing staff, the physiotherapist, and the speech pathologist for tracheostoma care. The speech pathologist also provided training on voice prosthesis care and swallowing. This education and training was discussed as a factor that supported participants’ adjustment in the home.

"I felt prepared by the speech pathologist for the management of my stoma, it made it easier. I left the house with a voice box and I returned home without one." P5

#### Phase Three: Just Get On With It (6 months post surgery )

Participants in this phase showed some acceptance that they were not back to the pre-morbid level but that they just had to be practical and get on with it. During this phase, no distress was reported by participants. It was during this phase that the participants were redefining what is normal to aid their adjustment. Participants were adjusting to their ‘new normal’ life; things were different now but there were also things that were going right in their life. They were starting to see gains with their communication and independence with activities of daily living and they were starting to have a more positive general outlook. Below are two participants’ descriptions of life 6 months post-laryngectomy:

"I’ve told myself I’ve got to live with it…I can’t do anything about it….get on with it….I’ve got a beautiful wife that pushes me…I know my life is different but I’ve got to get over that different. Just get on with life as normal." P4

"Things have improved. My weight has improved. My outlook has improved. I feel more positive. I’m getting everything to work, like my speech." P2

Adjustment in this phase was focused on the long-term effects of life post-laryngectomy and redefining the pathway of the ‘new normal’. Participants described how their tracheoesophageal speech was still impacting their communication with family members and in social settings, but that they were getting on with life. They had accepted their ‘new normal’ life:

"Life is alright…Socially it’s a little bit different as it stops me from chatting a lot."P3

"I had to adjust to going out and trying to communicate with others who couldn’t understand me. I can’t communicate with my partner at times and I try and I try and my voice doesn’t work." P2

In earlier phases, especially Phase 1, participants struggled with the idea of dependence. Now, in this later phase, participants highlighted more independence as being a defining factor in feeling that life was becoming more normal again. Participants were becoming more independent with their tracheostoma care and managing their voice prosthesis:

"I really don’t need help. I manage myself. I’m an independent person." P2

"Friends want to help, they always ask can we do anything but I’m very independent." P4

## Discussion

This is the first prospective qualitative study exploring the lived experience of adjustment within the first 6 months following a laryngectomy. It was evident that there were moderate levels of distress in phase one of adjustment; however, there was little to no distress seen in later phases. The low level of distress may be attributed to the participants’ coping skills and adjustment.

The study shows that adjustment to cancer and having a laryngectomy is not a single event but rather a series of ongoing coping responses to the various life changes, matching the process of adaptation as described by Brennan [18]. The process of psychosocial adjustment and the sequence of stages described by participants in this study align with the grief process in that participants described an initial period of shock, which was followed by significant distress and concluded with acceptance of their situation [19].

For participants, managing, learning from, and accommodating change started as early as the diagnosis of cancer and being informed of the need for life-saving laryngectomy surgery. The time frame between diagnosis and the surgical intervention was often as short as 2 weeks. During this time, participants were processing the shock of diagnosis as well as preparing for their surgery. Participants accommodated change by accepting their need for a laryngectomy, often with a focus on their family and loved ones. The distress at this point may have impacted on participants’ ability to accommodate change. One participant in the study experienced such high levels of distress that they contemplated suicide. This has been discussed in previous literature; for example, Parker et al. [20] noted that persistent high levels of distress can lead to poor adjustment. Thus, this study supports the need for psychosocial support for individuals prior to laryngectomy surgery. Furthermore, when psychological supports focus on distress in these early stages, it can contribute to better and more personalised counselling and support for patients [21].

Once the surgery was completed, participants discussed the hospital experience as contributing to their capacity to ‘manage, learn from and accommodate’ the changes they were experiencing. This was experienced in both positive and negative ways. On the negative side, participants saw the recovery period in hospital as reducing control and removing them from ‘normal life’ and routines, while they dealt with physical disfigurement and healing. Participants described their hospital stay as too long, and they reported on the frustration they felt during their hospital stay and what they missed. On the other hand, the hospital admission was also described as a period that assisted adjustment to life after cancer. Experiences that facilitated adjustment for participants were from the support, confidence building, kindness, and positivity they experienced from staff and family. Participants acknowledged that any loss of confidence was supported by caring nurses, doctors, and allied health staff, who were encouraging their adjustment to their altered breathing, voice, and swallowing. Participants also discussed the value of being understood as a person by health care professionals and how this aided their adjustment. The health care professionals understood their condition and current needs. Being supported by health care professionals and family adds to participants’ sense of confidence and resilience [14]. The care of health professionals was crucial to the participants’ adjustment. Research has previously pointed to health care professionals having a significant and profound influence on the lives of people with laryngectomy, because they are knowledgeable, skilled, and empathetic, and support patients over time [14].

In this first phase of adjustment, participants realised that they required a lot of support from family or friends. Family involvement and support have been identified in other studies as critical for participants’ success upon returning home [9, 22]. Given the numerous challenges facing an individual with cancer, support from caregivers is essential to facilitate successful coping and adjustment [9, 23, 24]. A theme of dependency on others was reported in a study by van Sluis et al. [25], where participants greater than 1 year post-laryngectomy reported a continuous dependency on their social network and on health care professionals.

The present participants indicated returning home was being a pivotal point in the adjustment process post-laryngectomy. Home represented a sanctuary, freedom, no pressure, and normal routine; at home, they could start to accommodate the changes that they were experiencing. This idea of discharge home being pivotal is supported by previous studies showing that patients find comfort in the familiar surroundings of home and being with family following discharge from hospital [9, 26]. Individuals living with laryngectomy face many obstacles in their journey and being in the home environment aids that transition and adjustment to the life changes. In the present study, the close involvement of family and friends in the participants’ home life was crucial to their successful adjustment. Bickford et al. [14] also highlighted that empathy and timely support from family and friends were highly valued by individuals with laryngectomy.

A major challenge for the present participants’ adjustment to an altered self-identity was accepting that their voice was permanently changed. In van Sluis et al. [25], participants greater than 1 year post-laryngectomy described learning to speak again as a physical and emotional challenge. Coping with their altered speech was a daily reminder to the present participants that life was never going to be the same again. Adjustment in this period was less about accommodating the change into their lives but accommodating their lives to the change. Decreased communication ability after total laryngectomy makes the adjustment particularly difficult and confronting for those individuals’ self-identity; this is particularly so for female tracheoesophageal speakers, who perceive themselves, and are perceived by others, as having less acceptable speech compared to male tracheoesophageal speakers (Bickford et al., 2018).

Key factors that supported participants regaining their independence included the importance of being in the home environment, support from others, and keeping socially connected. Living with the physical and psychological difficulties post-laryngectomy has been described in previous studies as a continuous struggle [14, 26]. This life-saving surgery has a profound and long-lasting impact on an individual’s self-identity [14]. There is also evidence to support the persistent vulnerability in the long term, including dependence on others, stigma-related problems, and returning to social and work-related activities [25].

### Implications for Practice

The findings suggest that there are many individual differences in how people adjust to the lived experience of having a laryngectomy. A patient-centred approach to education and supportive care is essential prior to and following treatment [25]. Moderate distress levels were shown in phase one of adjustment; therefore, it is vital that supportive care is provided in the pre and acute post operative setting by health care professionals, particularly dedicated support from social workers and psychologists. It is important that all patients are seen for psychosocial support in the diagnosis and early acute phase of their recovery to reduce the levels of distress experienced and to attempt to determine what factors are contributing to the distress. Through understanding the relationship between distress and modifiable psychosocial factors, tailored interventions for individuals can reduce distress and maximise quality of life [21].

The study findings highlighted that early training of patients to increase their independence with tracheostoma care and voice prosthesis use is vital in assisting with earlier adjustment post-laryngectomy. Training supportive family or friends during a patient’s acute inpatient admission in how to promote earlier independence with care is also of high importance. Emotionally supportive relationships set the stage for positive adjustment [27]. There is also value in earlier training of patients post operatively to cope with their new tracheostoma, equipment needs, and voice prosthesis care, as this can impact positively on an earlier discharge from hospital. The significance of returning home was clearly a positive factor impacting adjustment and needs to be foremost in the minds of all health care professionals working with people immediately post-laryngectomy. The earlier the person returns home, the better off they will be, as it may contribute to earlier adjustment.

Supporting patients to independently manage their new self is also important. The physical changes of having a tracheostoma and a new way of speaking using tracheoesophageal speech impacted participants’ sense of identity. Health care professionals need to be mindful of supporting activity limitations and participation restrictions that arise from these changes to self-identity. Psychosocial adjustment post-laryngectomy can be impacted by the individuals’ coping response to the physical, communication, and psycho-emotional changes that occur, and earlier identification and acceptance of these is crucial for acceptance of their altered selves [22].

### Limitations

While the study sample is small and recruited from a single tertiary hospital, this research provides an in-depth view of lived experiences of participants’ adjustment to laryngectomy over time. The authors acknowledge potential outcome bias, as all participants had family support and most resided in metropolitan areas of Australia. Future research should explore the experiences of individuals without family support or those living in regional or remote areas, as their recovery and adjustment needs may differ. Additionally, further studies should examine the education and support needs of family members and caregivers to better facilitate adjustment and ongoing rehabilitation after laryngectomy.

The research team recognises the importance of reflexivity, analytic transparency, and adherence to qualitative reporting standards. Alternative qualitative study designs—such as longitudinal or explicitly reflexive analytic approaches—may allow for deeper theoretical exploration of meaning-making, identity reconstruction, and reflexive positioning over extended periods of recovery. The present study was intentionally designed as a clinically oriented, applied multimethod investigation focused on early post-laryngectomy adjustment within a defined postoperative timeframe. As such, it was not intended to function as a methodological exposition of reflexive thematic analysis nor as a theory-building qualitative study. These alternative approaches are well suited to addressing complementary research questions in this setting and will inform the design of future research building on the findings reported here.

## Conclusion

This study is the first to explore the process of adjustment within the first 6 months following laryngectomy. Participant interviews suggest that adjustment is a unique process that occurs gradually over time. Results from this study will inform clinical care with emphasis on early psychosocial support from a psychologist and/or social worker along with ongoing multidisciplinary support and education.

## Data Availability

No datasets were generated or analysed during the current study.
